# Sexually Transmitted Diseases: from HPV to HTLV - clinical profile and associated factors*

**DOI:** 10.1590/abd1806-4841.20153663

**Published:** 2015

**Authors:** Fabíola Suris da Silveira, Renan Rangel Bonamigo

**Affiliations:** 1 Universidade Federal de Ciências da Saúde de Porto Alegre (UFCSPA) – Porto Alegre (RS), Brazil.; 2 Hospital Nossa Senhora da Conceição – Porto Alegre (RS), Brazil.; 3 Ambulatório de Dermatologia Sanitária da Secretaria de Saúde do Estado do Rio Grande do Sul – Porto Alegre (RS), Brazil.

**Keywords:** Sexually Transmitted Diseases, Risk Factors, Human T-lymphotropic virus type I, Human T-lymphotropic virus type II

## Abstract

The Brazilian Ministry of Health recommends the performance of serological tests in patients with clinical signs of Sexually Transmitted Diseases. However, data are lacking to affirm the necessity of testing these patients for human T-lymphotropic virus type 1 or type 2. This is a cross-sectional study with 120 patients seen at the Sexually Transmitted Diseases unit of the Sanitary Dermatology Outpatient Clinic of Rio Grande do Sul. The serum from none of the patients was positive for human T-lymphotropic virus type 1 or type 2. Viral warts were the most frequent diagnosis. Drug use was confirmed as a risk factor and high educational levels were found to be a protective factor against Sexually Transmitted Diseases.

According to the recommended by the Ministry of Health of Brazil, when people with clinical signs of Sexually Transmitted Diseases (STDs) seek health services, they are tested for human immunodeficiency virus (HIV), syphilis, hepatitis B virus and hepatitis C virus. However, according to the local epidemiological context, there is not enough data to affirm the need of testing for human T-lymphotropic virus type I and II (HTLV-1 and -2).

The serologic diagnosis of HTLV-1 and -2 infections could make the identification of new cases of subclinical infection possible and potentially aid in the management of possible clinical manifestations that are attributable to virus. Moreover, it could serve as a warning sign regarding the importance of STD and HTLV coinfection.^1^ This study aimed to: evaluate the relevance of performing a diagnostic screening using a high sensitivity assay for detecting HTLV infection in patients with STDs; and present the clinical and epidemiological profile of a sample of patients with STDs.

This is a contemporary cross-sectional study with control. We evaluated patients covered by the Unified Health System and seen at the STD unit of Sanitary Dermatology Outpatient Clinic of the State of Rio Grande do Sul.

This study was approved by the Research Ethics Committee of the Federal University of Halth Sciences of Porto Alegre and all patients signed an informed consent form. We used convenience sampling. Categorical data were analyzed using the chi-square test (with or without Yates' correction) or Fisher's exact test. Comparing the groups, the unadjusted odds ratios (ORs) with 95% confidence intervals (95% CI) were calculated by measuring the association between the variables of interest. Logistic regression was performed to adjust the estimates of the potentially associated factors. A value of p <0.05 was considered statistically significant, with 80% power.

Inclusion criteria were: patients with clinical symptoms of sexually transmitted infections, classified according to the syndromic approach into genital ulcers, urethral discharge, vaginal discharge, pelvic pain and genital warts. All patients were tested for HTLV-1 and -2 using the ELISA test (EnzymeLinkedImmuno-SorbentAssay). A control group was formed in order to benchmark the results of the HTLV serology (blood donors of the blood bank of the Hospital Santa Casa de Porto Alegre). This happened at the same period as data collection, in August 2011 and April 2012.

The variables studied were age, gender, educational level, city of residence, syndromic diagnosis of STDs, serology for HTLV-1 and -2, serology for syphilis, use of drugs and/or alcohol, history of STD/HIV, history of previous serological tests for STDs, condom use, having a steady sexual partner or multiple sexual partners, and partner's history of STD. Tables 1 and 2 show the main study data.

**Table 1:** Frequency distribution of the variables according to the syndromic approach to STD diagnosis

		Genital ulcer	Discharges*	Genital warts	Pelvic Pain	Total	p
History of HIV	Yes	6	3	5	0	14	0.253§
	No	21	30	53	3	107	
Drugs use	Yes	15	25	48	3	91	0.039§
	No	12	8	10	0	30	
Condoms use	Always	10	10	19	0	39	0.282§
	Sometimes	12	18	32	1	63	
	Never	5	5	7	2	19	
Steady partner	Yes	18	18	36	1	73	0.593§
	No	9	15	22	2	48	
Multiple partners	Yes	8	13	16	1	38	0.702§
	No	19	20	42	2	83	
Partner with STD	Yes	5	9	10	0	24	0.433§
	No	12	11	33	2	58	
	Does not know	10	13	15	1	39	

* the sum of the urethral and vaginal discharges.

§ Pearson's chi-square test

**Table 2:** Clinical and socio-demographic profile of patients with STD (patients treated at the Sanitary Dermatology Outpatient Clinic of the State of Rio Grande do Sul, 2011 and 2012)

	N	%			N	%
**Gender**				**Use Marijuana**		
Male	68	56.7 %		Yes	11	9.2%
Female	52	43.3 %		No	109	90.8%
						
**Educational level**				**Use cocaine**		
None	2	1.7%		Yes	3	2.5%
1-3 years	7	5.8%		No	117	97.5%
4-7 years	25	20.8%				
8-11 years	71	59.2%		**Use crack**		
Incomplete higher education	11	9.2%		Yes	1	0.8%
Complete higher education	4	3.3%		No	119	99.2%
						
**City of residence**				**Ingest alcohol**		
Porto Alegre, RS, Brazil.	98	81.7%		Daily	3	2.5%
Others	22	18.3%		Sometimes	78	65%
				Never	39	32%
**SEROLOGY**						
**Current serology for HTLV**				**OTHER FACTORS**		
Negative	119	99.2%		Use condom		
Inconclusive	1	0.8%		Always	40	33.3%
				Sometimes	62	51.7%
**Serology for Syphilis**				Never	18	15%
Positive	12	10%				
Negative	108	90%		**History of HIV**		
				Yes	14	11.7%
**SYNDROMIC APPROACH STDs**				No	106	88.3%
Vaginal discharge						
Yes	19	15.8%		**History of previous STD**		
No	101	84.2%		Yes	40	33.3%
				No	80	66.7%
**Genital ulcer**						
Yes	27	22.5%		**Were already tested for STD**		
No	93	77.5%		Yes	77	64.2%
				No	43	35.8%
**Urethral discharge**						
Yes	3	2.5%		**Havea steady partner**		
No	117	97.5%		Yes	73	60.8%
				No	47	39.2%
**Pelvic Pain**						
Yes	14	11.7%		**Have multiple partners**		
No	106	88.3%		Yes	36	30%
				No	84	70%
**Genital warts**						
Yes	58	48.3%		**Partner with STD**		
No	62	51.7%		No	57	47.5%
				HIV	6	5.0%
**DRUGS AND ALCOHOL USE**				HTLV	1	0.8%
Smoke cigarettes				HPV	11	9.2%
Yes	40	33.3%		Other	8	6.7%
No	80	66.7%		Does not know	37	30.8%

n=120

Mean age 30.9 (±13.3)

Minimum age = 14 years

Maximum age = 77 years

120 patients were included in the study. 56.7% were male. The mean age was 30.9 (±13.3) years. The serum from none of the patients was positive for HTLV-1 and -2. 10% of patients had positive serology for syphilis. A higher level of education of eight years and more was associated with an increased use of condoms, when compared with lower levels of education (p <0.05). Drug use was associated with positive serology for HIV, genital ulcers and urethral discharge (p <0.05).

Genital warts were the most frequently diagnosed STD (48.3%), revealing the importance of infections caused by papilloma virus (HPV).

The mean seroprevalence of HTLV-1 and -2 in Brazil is 0.5%, with higher prevalence in the North and Northeast and lower prevalence in the South of the country.^2,3^ In Porto Alegre, the prevalence among blood donors is 0.17%.^2^ On the other hand, the seroprevalence of syphilis (10%) was very high, when compared with blood donors used as controls (0.5%).

We found a high consumption of licit and illicit drugs (over 75%) among the participants. There was a significant association between all STDs and the use of at least one type of drug.

A study conducted with adolescents in Ceará demonstrated the influence of alcohol on risk behavior STDs.^4,5^ In our study, the use of drugs was associated with multiple sexual partners and with urethral discharge.

High levels of HTLV infection relate to the use of drugs and to relationships with drug users.^6,7^ A study conducted in 2005 showed that 2.4% of HIV patients were seropositive for HTLV. The use of intravenous cocaine was the most important risk factor.^7^

It is known that low educational levels are associated with a higher prevalence of HTLV-1 and syphilis.^8,9^ In this study, most participants had an educational level of eight years study or more, and this was associated with the frequent use of condoms. Genital warts were more prevalent in individuals with higher educational levels. This finding may be related to greater concern about health and an increased demand for health care.

In conclusion, viral warts were the most frequently syndromically diagnosed STD and no coinfections with HTLV were observed. The inclusion of a diagnostic screening for detecting HTLV-1 and -2 in patients with STDs does not seem to be relevant for patients seen in a referral center in Porto Alegre. It is noteworthy that the use of drugs constitutes a major risk factor for STDs and the high educational levels are possibly a protective factor against STDs.
